# Vertebral Artery Injury in Cervical Spine Fracture Dislocation: An Observational Study in a Tertiary Care Center

**DOI:** 10.7759/cureus.57774

**Published:** 2024-04-07

**Authors:** Md. Ziaul Hasan, Gururaj M Sangondimath, Md. Shah Alam, Fazal Rehman T, Vishal Singh, Prarthan C Amin, Pratik Patel

**Affiliations:** 1 Orthopedic and Spine Surgery, National Institute of Traumatology and Orthopedic Rehabilitation (NITOR), Dhaka, BGD; 2 Spine Services, Indian Spinal Injuries Center, New Delhi, IND; 3 Spine Surgery, Bangladesh Spine & Orthopaedic Hospital, Dhaka, BGD; 4 Spine Surgery, Indian Spinal Injuries Center, New Delhi, IND; 5 Spine Surgery, Sri Balaji Action Medical Institute, New Delhi, IND; 6 Spine Services, Indian Spinal Injuries Center, Ahmedabad, IND; 7 Spine Surgery, Viroc Hospital, Vadodara, IND

**Keywords:** magnetic resonance imaging, vertebral artery injury (vai), spinal cord injury, fracture dislocation, traumatic cervical spine injury

## Abstract

Background: The incidence of traumatic vertebral artery injury (VAI) associated with cervical spine trauma varies widely in published trauma series. The primary aim of this study was to determine the incidence of traumatic VAI in patients who suffered cervical spine injuries by means of routine magnetic resonance imaging, and the secondary objective was to identify any associations with injury mechanism, level of injury, and neurologic injury severity.

Materials and methods: A retrospective review was conducted on 96 patients who suffered cervical spine fracture dislocation with or without an associated spinal cord injury (SCI) in Indian Spinal Injuries Center (ISIC), New Delhi, India from January 2013 to April 2023. Cervical magnetic resonance imaging (MRI) was used to diagnose VAI. Patient’s age, sex, cervical injury level, mechanism of injury, neurologic level of injury, association with foraminal fracture, facet dislocation, and clinical sequelae of vertebral artery injury were analyzed.

Results: In this study, of 96 patients who met the inclusion criteria, 18 patients (18.75%) had VAI on the MRI study. Thirteen (72.22%) of the eighteen patients had right-sided injuries, four (22.22%) had left-sided injuries, and one (5.55%) had bilateral injuries. There was an associated SCI in every VAI patient. VAI was significantly more common in patients who had ASIA A (61%, n = 11) and ASIA B (22%, n = 4) injuries, and no VAI was noted in neurologically intact patients (p<0.001). The incidence of VAI was higher in the flexion distraction type of injury (n = 12, 66%). The most commonly involved cervical spine injury level was C5-C6 (27%, n = 5), followed by 22% (n = 4) at C4-C5 and C6-C7 levels. About 27.8% (n = 5) of VAI was associated with foraminal fractures, and 72% (n = 13) of VAI was associated with facet dislocations, of which 44% (n = 8) were bifacetal and 28% (n = 5) were unifacetal dislocations. On clinical symptoms, only one (5.56%) patient had a headache, and 17 (94.4%) had no clinical features due to VAI.

Conclusion: The incidence of traumatic vertebral artery disease is not very uncommon and requires careful and meticulous screening and management. Otherwise, complications like pseudoaneurysm, neurologic deficit, late-onset hemorrhage, infarction, and death can happen. Mostly, it is associated with high-velocity injuries and neurological injuries. MRI can be used as a good screening tool, which can be aided by a CT angiogram or digital subtraction angiography for confirmation. Proper pre-operative evaluation of vascular injury in cervical spine fracture dislocation is very important for patient counseling, patient management, and surgical planning.

## Introduction

A cervical spine injury is a potentially life-threatening as well as neurologically disabling injury [1]. Association with vertebral artery injury (VAI) may make things more challenging at times. It has been observed that the incidence of VAI following cervical spinal trauma ranges from 25% to 46% in the published spine literature [2].

Possible causes of this variable incidence rate are mostly due to the asymptomatic nature of VAI, owing to its bilateral distribution and strong collaterals. The vertebral artery, emerging from either the subclavian or innominate artery, makes its entry into the transverse foramen at the C6 level. Initially positioned anterior to the transverse foramina of the 7th cervical vertebra, it traverses through a sequence of foramina, advancing toward the base of the axis. Subsequently, the artery curves posteriorly, entering the foramen transversarium located in the posterolateral section of the ring of atlas, before proceeding into the foramen magnum. Here, it joins the vertebral artery from the contralateral side, resulting in the formation of the basilar artery. This artery is pivotal in delivering blood to the brainstem and cerebellum [3].

VAI most commonly occurs in its second segment as it passes through the sub-axial spine, as a consequence of its relatively rigid positioning within the foramen transversarium. In cases of severe cervical spine injuries, especially when there is an associated subluxation of the facet joint or a fracture of the foramen transversarium, there is a greater incidence of VAI.

Unilateral injury to the vertebral artery seldom causes symptoms if the collateral from the contralateral vertebral artery and also from the posterior inferior cerebellar artery is sufficient [4]. Bilateral VAI can also present without any symptoms, but it has the potential to cause neurological deficits and, in extreme cases, even death [5]. Magnetic resonance imaging (MRI) is recognized as the gold standard procedure for evaluating cervical spine injuries, providing detailed information about the status of cord and other soft tissue structures. To detect VAI, fat-suppressed T1-weighted, and T2-weighted sequences are studied in either the oblique or axial planes using a 1.5 Tesla MRI machine.

The primary aim of our study was to determine the incidence of traumatic VAI in patients who suffered cervical spine injury by means of routine MRI, and the secondary objective was to identify any associations with the injury mechanism, level of injury, and neurologic injury severity and to verify the diagnostic value of MRI for the diagnosis of VAI.

## Materials and methods

This research was carried out at a single tertiary care center. A retrospective review was conducted on 96 patients who suffered cervical spine fracture dislocation with or without an associated spinal cord injury (SCI) in our institute from January 2013 to April 2023. Inclusion criteria included patients with cervical spine fractures/subluxations occurring between C1 and C7 with or without neurological deficits. Patients with penetrating cervical spine or vertebral artery injuries were excluded from the study. Patients with cervical spine injuries were classified using the Allen and Ferguson classification system for the subaxial spine after an extensive review of the X-ray and MRI results. Patients with supra-axial injuries were classified based on the nature of specific bony component injuries.

**Figure 1:**
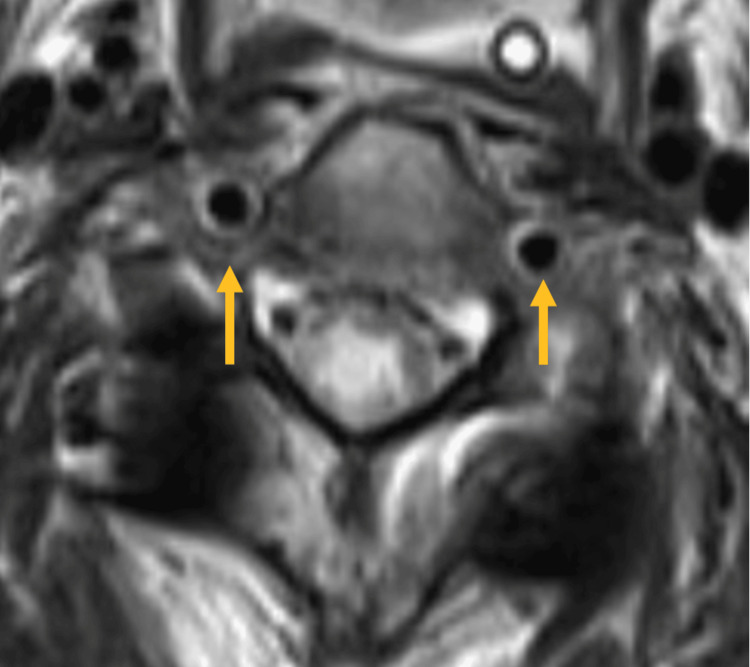
Presence of normal flow void on both sides of the vertebral artery

**Figure 2:**
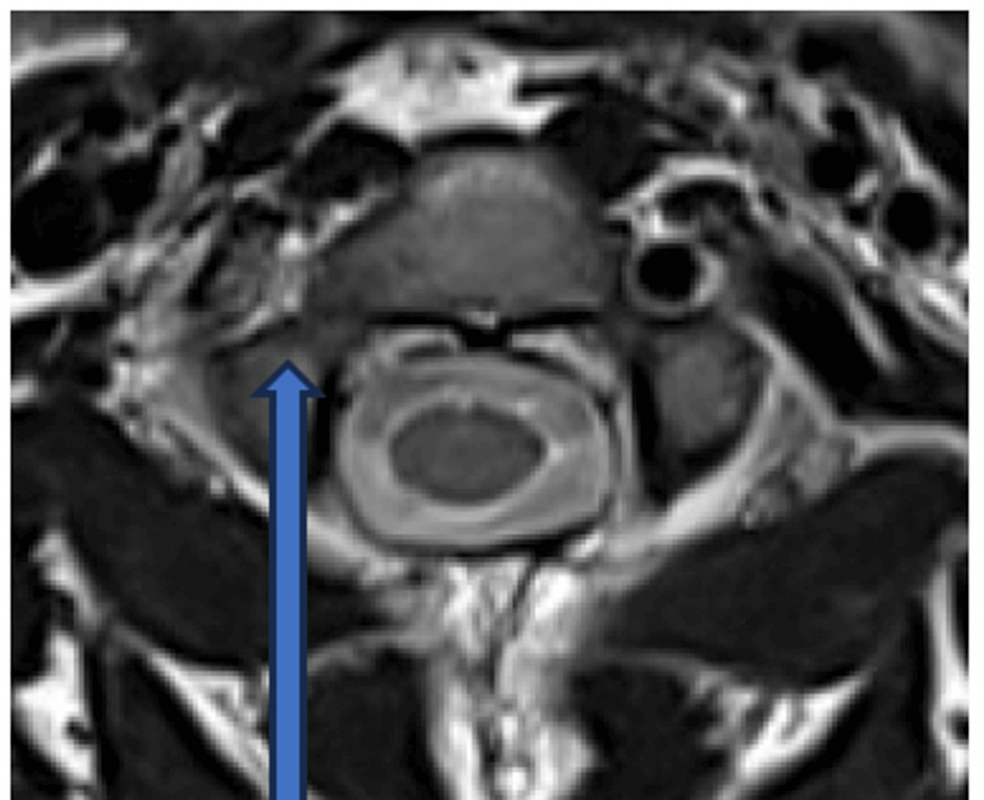
Absence of normal flow void in the right-sided vertebral artery

All MRI scans were evaluated for signal changes in the axial T1W and T2W sequences. The combination of T1-weighted sequences with fat saturation and T2 axial sequences is effective and ideal for detecting VAI. This can also identify intramural hematoma, thrombosis, and dissection, particularly in the V2 and V3 segments of the vertebral artery. These intramural pathologies manifest as hyperintense areas with a "crescent" shape. This appearance is due to the eccentric, circular, hyperintense residual arterial lumen as a result of flowing blood in the T1 fat-suppressed sequence. The normal vertebral artery is represented by the presence of flow void on both sides in the T2 sequence (Figure 1), and the injury presents as crescentic hyperintensity encompassing the VA wall, accompanied by a complete absence of flow void (Figure 2).

The mechanism of injury, level of injury, and clinical presentation of patients were studied as per hospital records and imaging. Neurological involvement was assessed by ASIA scoring. Patient’s age, sex, cervical injury level, mechanism of injury, neurologic level of injury, and American Spinal Injury Association (ASIA) grade and clinical sequelae of vertebral artery injury were analyzed. In this study, we also analyzed the relationship between facet dislocation and VAI, foraminal fracture and VAI, and the association of neurological injury with VAI.

## Results

The mean ±SD age was 42.23 ±15.30 years (Table 1).

**Table 1:** Age of the participants (n=96) Data are expressed as mean ±SD

Variables	Mean ±SD	Range
Age (years)	42.23 ±15.30	14-74

In this study, among 96 patients, 77 (80.2%) were male and 19 (19.8%) were female (Table 2).

**Table 2:** Sex distribution of the participants (n=96) Data are expressed as frequency (percent of cases)

Sex	Frequency	Percentage
Male	77	80.2%
Female	19	19.8%
Total	96	100%

**Figure 3:**
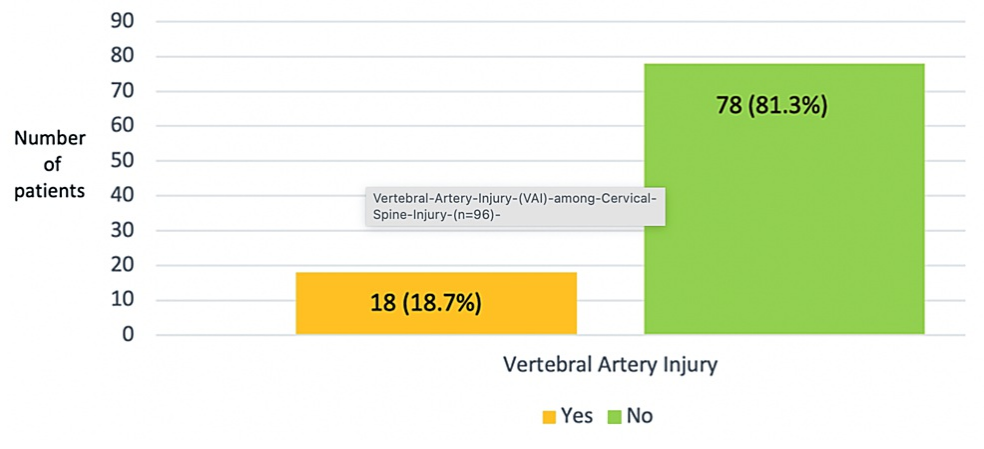
Vertebral artery injury (VAI) among cervical spine injury (n=96)

Of the 96 patients who met the inclusion criteria, 18 (18.75%) had VAI in the MRI study (Figure 3).

**Figure 4:**
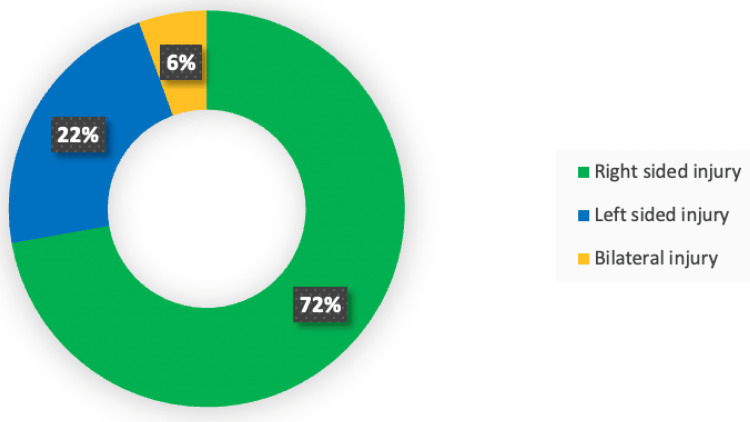
Side of VA injury (n=18) VA: Vertebral artery

Thirteen (72.22%) of the 18 patients had right-sided injuries, four (22.22%) had left-sided injuries, and one (5.55%) had bilateral injuries (Figure 4).

There was an associated SCI in every VAI patient. VAI was significantly more common in patients who had ASIA A (61%) and ASIA B (22%) injuries (p<0.001), and no VAI was noted in neurologically intact patients (Table 3).

**Table 3:** Neurological injury (ASIA) associated with VAI (n=96) Data are expressed as frequency (percentage); the p-value was derived from the chi-square test p < 0.001 indicates statistically highly significant; hs: highly significant VAI: Vertebral artery injury

ASIA Grade	VAI		p-value
	Yes	No	
A	11 (61.1%)	9 (11.5%)	<0.001 (hs)
B	4 (22.2%)	20 (25.6%)	
C	3 (16.7%)	33 (42.3%)	
D	0 (0%)	14 (17.9%)	
E	0 (0%)	2 (2.6%)	
Total	18 (100%)	78 (100%)	

The most commonly involved cervical spine injury level associated with VA (n=18) was C5-C6 (27%), followed by 22% in both C4-C5 and C6-C7 levels (Table 4).

**Table 4:** VAI in the associated level of injury of the study subjects (n=18) Data are expressed as frequency (percentage); the p-value was derived from the chi-square test p > 0.05 indicates statistically non-significant; ns: not significant

Level of injury	VAI		p-value
	Yes	No	
C1-C2	0 (0%)	14 (17.9%)	0.179 (ns)
C2 fracture	0 (0%)	2 (2.6%)	
C2-C3	0 (0%)	2 (2.6%)	
C3-C4	1 (5.6%)	4 (5.1%)	
C4-C5	4 (22.2%)	7 (9.0%)	
C5-6	5 (27.8%)	17 (21.7%)	
C6	2 (11.1%)	3 (3.8%)	
C6-7	4 (22.2%)	20 (25.6%)	
C7	0 (0%)	2 (2.6%)	
C7-T1	0 (0%)	4 (5.1%)	
Whiplash	2 (11.1%)	3 (3.8%)	
Total	18 (100%)	78 (100%)	

The most common mechanism of injury to the cervical spine in the total study population was flexion dislocation, which was 53%, n = 53. The incidence of VAI was higher in the flexion distraction type of injury (n = 12, 66%), followed by the distractive extension (22%, n = 4), and acceleration-deceleration injury (11%, n = 2) (Table 5).

**Table 5:** Classification of injury associated with VAI of the patients (n=96) Data are expressed as frequency (percentage); the p-value was derived from the chi-square test p > 0.05 indicates statistically non-significant; ns: not significant VAI: Vertebral artery injury

Classification	VAI		p-value
	Yes	No	
Acceleration-deceleration	2 (11.1%)	3 (3.8%)	0.179 (ns)
Axial compression	0 (0%)	14 (17.9%)	
C1 fracture	0 (0%)	2 (2.6%)	
C1 Pars fracture	0 (0%)	2 (2.6%)	
Compressive flexion	0 (0%)	7 (9.0%)	
Destructive extension	4 (22.2%)	17 (21.7%)	
Flexion distraction	12 (66.7%)	3 (3.8%)	
Lateral flexion	0 (0%)	20 (25.6%)	
Vertical compression	0 (0%)	2 (2.6%)	
Total	18 (100%)	4 (5.1%)	

About 27.8% (n = 5) of patients in the VAI group had an associated foraminal fracture, whereas 11.5% (n = 9) of the non-VAI group had an associated foraminal fracture (Table 6).

**Table 6:** Foramen fracture among the patients (n=96) Data are expressed as frequency (percentage); the p-value was derived from the chi-square test p > 0.05 indicates statistically non-significant; ns: not significant VAI: Vertebral artery injury

Foraminal Fracture	VAI		p-value
	Yes	No	
Yes	5 (27.8%)	9 (11.5%)	0.078 (ns)
No	13 (72.2%)	69 (88.5%)	
Total	18 (100%)	78 (100%)	

Here, 44.4% (n = 8) patients in the VAI group and 23.1% (n = 18) patients in the non-VAI group had an associated bifacetal dislocation, 27.8% (n = 5) patients in the VAI group and 14.1% (n = 11) patients in the non-VAI group had an associated unifacetal dislocation, and 27.8% (n = 5) patients in the VAI group and 62.8% (n = 49) patients in the non-VAI group had no associated facetal dislocation (p = 0.026) (Table 7).

**Table 7:** Association with facet dislocation (n=96) Data are expressed as frequency (percentage); the p-value was derived from the chi-square test p < 0.05 indicates statistically significant; s: significant VAI: Vertebral artery injury

Facet dislocation	VAI		p-value
	Yes	No	
Bifacetal	8 (44.4%)	18 (23.1%)	0.026 (s)
Unifacetal	5 (27.8%)	11 (14.1%)	
No	5 (27.8%)	49 (62.8%)	
Total	18 (100%)	78 (100%)	

On clinical symptoms, only one (5.56%) patient had a headache, and 17 (94.4%) had no clinical features due to VAI; 83.3% (n = 15) patients had a complete motor injury (ASIA A or B), and 17.7% (n = 3) patients had an incomplete motor injury (ASIA C, D, or E).

## Discussion

According to research, the rate of cervical spine injury complications caused by VAI is between 25% and 46%. The rate of complications caused by cerebral infarction resulting from VAI is between 0% and 54%, and the fatality rate is between 0% and 18% [6-8]. Furthermore, as a result of VAI, 7%-20% of individuals with cervical spine injuries experience vertebral artery occlusion [8,9]. The objective of the study was to determine the frequency of traumatic vertebral artery injury in patients with cervical spine trauma using routine magnetic resonance imaging and identify associations with age, sex, mechanism of injury, level of injury, and severity of neurologic injury.

Injury to the vertebral artery was found in 18 (18.7%) of the 96 cases of cervical spine injury patients, which were diagnosed by a loss of the normal flow void seen on the axial cuts of the MRI. Out of the patients, 17 (94.44%) had no symptoms at all from their VA damage, and only one (5.5%) patient reported having an occipital headache. In a study by Rathod et al. (2020), the incidence of VAI was 14.75% (n = 61) [1]. Another study done by Giacobetti et al. found 19.7% (n = 61) VAI in their patients [9]. A study done by Strickland et al. showed the patient did not have any cranial symptoms despite his bilateral VAI [10]. Of the 68 patients with a confirmed cervical spine fracture, five (7.35%) were diagnosed with VAI in another study by Sheppard et al. [11]. Miller et al. and Biffl et al. also presented the high incidence of VAI in blunt cervical spine trauma, ranging from 33% to 39% VAI incidence [12,13].

According to our observations, the C5-C6 level of cervical spine injuries was the most frequently involved, at 27% (n = 5), followed by the C4-C5 level and the C6-C7 level (22%, n = 4). This might be attributable to the most mobile junction of the cervical spine column being C5-C6 (12). This is similar to the findings of the study conducted by Giacobetti et al. [9].

The flexion destruction type of injury, which occurred 51 times (53%) in the research population, was the most frequent mechanism of cervical spine damage. The most frequent kind of VAI, accounting for 12 cases (66%), was flexion distruction, with distructive extension occurring in four cases (22%), and acceleration-deceleration type injuries occurring in two cases (11%). Rathod et al. (2020) reported that in the cohort of nine cases of VAI, eight patients had subaxial cervical spine injuries, of which seven were due to flexion-distraction injuries. The C5-C6 flexion-distraction injury was most commonly associated with VAI (four cases) [1]. 

The current case series demonstrates that five patients (27.8%) in the VAI group and nine patients (11.5%) in the non-VAI group both had concomitant foraminal fractures throughout the same period. In their study by Sheppard et al. (2020), two (40%) involved fractures extending into the transverse foramen, two (40%) involved subluxation of the vertebrae, and one (20%) involved both. In all five cases, these fractures resulted from high-energy injuries [11]. Biffl et al. showed 22% (n = 6) VAI was associated with foramen fracture [13]. The study done by Gupta et al. had 20 cases (42.5%) of VAI, and there was an extension of the fracture through the foramen transversarium [14]. Cothren et al. showed 26% (n = 18) associated foramen fractures in VAI patients [15].

In our series, eight (44.4%) cases of VAI were associated with bifacetal dislocation, 5 (27.8%) cases of VAI were associated with unifacetal dislocation, and another 5 (27.8%) cases of VAI weren’t associated with facet dislocation. Gupta et al. showed that 55% (n = 26) of their cases had an associated facet dislocation with or without a fracture [14].

In this study, of 96 patients who met the inclusion criteria, 18 patients (18.75%) had VAI on the magnetic resonance imaging study. Thirteen (69.4%) of the eighteen patients had right-sided injuries, four (23.32%) had left-sided injuries, and one (5.55%) had bilateral injuries. A study done by Parbhoo et al. found that 12 patients (25%) had vertebral artery injuries (one bilateral) [16]. Another study by Rathod et al. showed the incidence of VAI as 14.75% (n = 9) [1]. A study done by Mitha et al. found that VA abnormalities were present in 19.7% of cases [17].

In our study, 19 patients were female, and 77 were male among the 96 patients. The mean age was 42.43 years. Sheppard et al. described that there were 39 males (57.4%), 29 females (42.6%), and a mean age of 60.4 years in their study [11]. The mean age was 33.5 years, with 46 males and 15 females in their study group by Rathod et al. (2020) [1].

Of the VAI patients, 3 (17.7%) had motor incomplete injuries, and about 15 (83.3%) had motor full injuries (ASIA A or B). A study done by Torina et al. showed VAI was significantly more common in motor-complete patients (ASIA A and B, 20%) than in neurologically intact (ASIA E, 11%) cervical spine-injured patients [18]. So more severe neurological damage is associated with VAI.

Still, our study has some limitations. As it is a retrospective review, direct patient contact was not possible, and no follow-up could be done. Most importantly, no treatment relationship for VAI patients could be established. MRI is not the gold standard for diagnosing VAI, and in our study, VAI cases could not be confirmed by digital subtraction angiography (DSA) or CT angiography.

## Conclusions

The incidence of traumatic vertebral artery disease is not very uncommon and requires careful and meticulous screening and management. Otherwise, complications like pseudo-aneurysm, neurologic deficit, late-onset hemorrhage, infarction, and death can happen. Mostly, it is associated with high-velocity injuries and neurological injuries. MRI can be used as a good screening tool, which can be aided by a CT angiogram or DSA for confirmation. Proper pre-operative evaluation of vascular injury in cervical spine fracture dislocation is very important for patient counselling, patient management, and surgical planning.
